# CYP24A1 and CYP3A4 Levels, Renal, Hepatic Changes, and Incidence of Oxidative Stress in Tramadol-Alcohol Concomitant Misuse

**DOI:** 10.7759/cureus.36877

**Published:** 2023-03-29

**Authors:** Ebenezer O Dic-Ijiewere, Humphrey B Osadolor

**Affiliations:** 1 Medical Laboratory Science, University of Benin, Benin, NGA

**Keywords:** cytochrome enzyme, kidney, liver, alcohol, tramadol

## Abstract

Introduction

Long-term population-based research has demonstrated a link between heavy drinking and the prevalence of kidney disorders; similarly, alcohol abuse has long been recognized as one of the main causes of liver diseases. A recent trend of concomitant use of the opioid analgesic Tramadol and alcohol among young males in sub-Saharan Africa has emerged.

Aim and objectives

This study’s primary aim was to evaluate the incidence of concomitant use of alcohol and Tramadol among adult males, and observe the role of cytochrome p450 3A4 and CYP24A1 proteins and some oxidative stress indicators such as Malondialdehyde, lactate dehydrogenase, among study participants. The secondary aim was to evaluate the effect of alcohol and Tramadol concomitant use on Liver and kidney indices.

Methods

Our study population was male subjects with a history of Alcohol and Tramadol concomitant use. Liver enzymes, renal indices, oxidative stress markers, and CYP3A4 and CYP24A1 were determined from the serum of test and control participants. IBM Statistical Package for Social Sciences (SPSS) Statistics (version 21.0) was used to analyze the data obtained.

Result

One hundred and forty-two male subjects were included in this study. Eighty two (82) were males who admitted to abuse of Alcohol and Tramadol concomitantly for at least a year. The dose of Tramadol commonly used by Test subjects was 200 mg (43.9% of the test population), Tramadol users in the study population were largely Undergraduates (75.6% of Test participants). Gamma-glutamyl transferase and lactate dehydrogenase were significantly higher in Test subjects consuming Tramadol and alcohol combination (43.13±1.02 and 117.29±2.45, respectively) versus control (24.87±0.82; p=0.00 and 101.93±1.25; p=0.00). There was a significant decrease in serum bicarbonate levels of Test subjects (16.19±0.53) versus control (22.60±0.68; p=0.000). Cytochrome P450 24A1, was significantly lower in Test subjects (subjects consuming Tramadol and alcohol combination) (0.90±0.06; p=0.01), and significantly threefold higher in subjects with acute myeloid leukemia (AML) (5.16±0.5; p=0.00), when compared with values of non-drug/alcohol users that served as normal control (1.27±0.07).

Conclusion

The menace of Tramadol and alcohol concomitant abuse has taken a worrisome dimension in sub-Saharan Africa. In this study 77.4% of participants reported euphoria as reason for combining Alcohol and Tramadol, 6.5% claimed it was for faster pain relief and enhanced sexual performance or prolong penile erection was the response of 58.1% of the test participants. Findings of reduced CYP3A4 with Alcohol and Tramadol concomitant use could be associated with delayed drug inactivation and increased drug euphoric action.

## Introduction

Tramadol, a popularly prescribed opioid, is an effective analgesic agent for the treatment of moderately severe acute or chronic pain [1], although it can be obtained from the counter without a prescription in common drug stores. O-desmethyl-Tramadol the active product two to four times more potent than Tramadol is metabolized in the liver. Further, bio-transformation results in inactive metabolites, which are excreted by kidneys [1,2]. Studies have revealed that the combination of Tramadol and Alcohol could cause life-threatening or even fatal side effects. Using alcohol while administering Tramadol can result in liver and central nervous system damage [3]. Concomitant Alcohol and Tramadol intake, as well as intake of Alcohol-Tramadol cocktails, are a recently observed trend among young Nigerians [4,5].

The liver is the organ that takes the most harm from alcohol and drug consumption based on available information. Alcohol can be involved in the development of other diseases of the liver that are not specifically attributed to it and may interact with risk factors for other forms of liver disease. Alcoholic liver disease (ALD) is a blanket term under which conditions related specifically to the liver and alcohol use fall under. The most prevalent types of ALD are fatty liver, alcoholic hepatitis, and cirrhosis. Often, with heavy alcohol consumption, fatty liver disease progress to liver cirrhosis or hepatitis. Alcohol, one of the many factors that can compromise renal function, can intrude with order function directly, through acute or habitual consumption, or laterally, as a consequence of liver disorder [6]. Tramadol is metabolized in the liver via two main metabolic pathways involving Cytochrome P450 family 3 subfamily A member-4 (CYP3A4) and Cytochrome P450 family 2 subfamily D member-6 (CYP2D6) Iso-enzymes to form active O-desmethyl metabolite (M1 or ODT) or inactive N-desmethyl metabolite (phase I reactions) [7,8]. The involvement of Cytochrome P450 family 24 subfamily A member-1 (CYP24A1) in Tramadol and Alcohol metabolism is not known prior to this study.

The current trend of mixing or combining alcohol with analgesics such as Tramadol and Diclofenac to enhance the feeling of drunkenness and ecstasy is a worrisome trend that requires detailed research in order to establish the potential danger inherent in this act. Hence, this study aims to investigate the danger inherent in Alcohol and Tramadol concomitant use with respect to pathological impact on the liver and kidney.

## Materials and methods

This study was an observational cross-sectional study carried out between the period of December 2020 and January 2021 at the Pathology Multipurpose laboratory of the College of Medical Sciences of Ambrose Alli University, Ekpoma, Nigeria. Ethical approval was obtained from the Health Research Ethics Committee of Ambrose Alli University Ekpoma, Nigeria (NHREC/12/06/2013/014/19) before the study commenced, and informed consent was obtained from all study participants in their “sober state.”

The study population consisted of 142 males between the ages of 19 years to 30 years. Eighty two of them were subjects that admitted to Alcohol and Tramadol concomitant intake for at least a year (test group), while 60 healthy male subjects with no history of Alcohol and Tramadol intake were normal controls. Four subjects diagnosed with acute myeloid leukemia (AML) served as positive control for CYP3A and CYP24A1.

The test group were subjects that admitted to Alcohol and Tramadol concomitant intake for at least a year, and have used an alcohol-Tramadol cocktail within Seven days of questionnaire administration and blood sample collection. Young males with clinical illnesses, and those who sparingly use Alcohol and Tramadol concomitantly, were excluded from this study. Demographic characteristics including age, Occupation, type of alcohol used concomitantly with Tramadol, reasons for alcohol-Tramadol combination, and other drugs taken in combination with alcohol, were obtained with the aid of a questionnaire.

Alcohol and Tramadol concomitant use was identified using the following questions (Q) with responses in parenthesis: Q 1: Have you combined alcohol with Tramadol? (Yes/No) - Q 2: What brand of alcohol did you use with Tramadol? (Beer/Spirit/Wine/Gin).

Tramadol abuse was assessed using the following question. Q 3: “The Tramadol in question was not prescribed by a doctor”. Kindly note, that your response is very confidential. What is the dose of Tramadol used? (50 mg /100 mg /200 mg />200 mg).

 Demographic characteristics such as Occupation and reasons for alcohol-Tramadol concomitant use were assessed using the following questions: Q 4: What is your occupation? - Q 5: What is your reason(s) for Tramadol and alcohol concomitant use?

Renal function tests include Potassium, Sodium, Chloride, Bicarbonate, creatinine, and Urea; liver function tests include alkaline phosphatase, aspartate aminotransferase, alanine aminotransferase, gamma-glutamyl transferase (GGT) and lactate dehydrogenase, Oxidative stress markers such as malondialdehyde (MDA), serum catalase, reduced glutathione and superoxide dismutase, CYP3A and CYP24A1 were assessed. Potassium, sodium, chloride and bicarbonate were assessed with the AFT-800 electrolyte Analyzer (Payal Agencies, Vandora, India). Alkaline phosphatase, aspartate aminotransferase, alanine aminotransferase, gamma glutamyl transferase and lactate dehydrogenase, creatinine and urea were assessed with the use of SP-113 visible spectrophotometer (Axiom Solutions, Burstadt, Germany). Malondialdehyde, serum catalase, reduced glutathione and superoxide dismutase were measured with the Labtech UV-Vis Spectrophotometer UV 9100-Series (Labtech International Ltd, Sussex, England). CYP3A and CYP24A1 were assessed using enzyme-linked immunosorbent assay (ELISA) methods, made up of antibodies specific to CYP24A1 and CYP3A4 proteins, and Avidin-Horseradish Peroxidase conjugate specific for CYP24A1 and CYP3A4, respectively (Elabscience, USA; Catalog No: E-EL-H0377, E-EL-H037-96T) and measured with the TECAN infinite f50 (Tecan trading AG, Switzerland).

The commercially available IBM Statistical Package for Social Sciences (SPSS) Statistics© 2012, windows version 21.0 (IBM SPSS Statistics, Armonk, NY) was used to analyze the data obtained using a sample t-test. Frequencies and Percentages were applied for the description of categorical demographic data. Mean and standard deviation was used to describe distributed continuous data. Analysis of Variance (ANOVA) was used to analyze the differences in Serum CYP24A1 and CYP3A4 levels between Normal control, Test and AML or positive control. Mean values were considered significant at P-value <0.05.

## Results

Two hundred and forty nine (249) participants from 2020 to 2021 were administered questionnaires, which was reduced to 142 study subjects, after excluding underage males (<16 years), subjects that sparingly concomitantly use Alcohol and Tramadol together, and those positive for Hepatitis B and C surface antigen serology test. 60 participants that admitted to non-alcohol and non-Tramadol/alcohol combination use were used as normal control, while 82 participants that admitted to Alcohol and Tramadol concomitant use within Seven (7) days of questionnaire administration were sampled as Test subjects (participants). The alcohol type most consumed in combination with Tramadol by test participants was Beer (41.5% of the test subjects), followed by Spirit (29.3% of test subjects), Wine (24.3%) and Gin (4.9%). The dose of Tramadol commonly used by test subjects was 50 mg (6.1% of the test population), 100 mg (34.1% of the test population), 200 mg (43.9% of the test population) and >200 mg (15.9%). From the demography, Tramadol users in the study population were largely Undergraduates (75.6% of Test subjects), while the other 12.2% of the test participants were artisans. The reasons alluded to for combining Alcohol and Tramadol were Euphoria (77.4% of the test subjects), pain relief (6.5% of the test subjects), and enhance sexual performance or a prolonged penile erection which 58.1% of the test participants alluded to. Felvin and 3,4-Methylene Dioxy-methamphetamine (MDMA) (commonly referred to as molly, Kleenax, eve, love drug, moon rock, ecstasy), were the other drugs commonly consumed with alcohol, admitted to by the study subjects (Table 1).

**Table 1:** Sociodemographic characteristics of study subjects MDMA: 3,4-Methylenedioxymethamphetamine

	Control	Test
Have you combined alcohol with tramadol?		
Yes	0(0.0%)	82(100.0%)
No	60(100.0%)	0(0.0%)
Alcohol Types taken with tramadol		
Beer	0(0.0%)	34(41.5%)
Spirit	0(0.0%)	24(29.3%)
Wine	0(0.0%)	20(24.3%)
Gin	0(0.0%)	4(4.9%)
Oral Dose of tramadol commonly used		
50 mg	0(0.0%)	5(6.1%)
100 mg	0(0.0%)	28(34.1%)
200 mg	0(0.0%)	36(43.9%)
> 200 mg	0(0.0%)	13(15.9%)
Occupation		
Artisan	4(06.7%)	10(12.2%)
Civil servant	2(03.3%)	0(0.0%)
Graduate	2(3.3%)	0(0.0%)
Secondary	6(10.0%)	0(0.0%)
Undergrad	46(76.7%)	62(75.6%)
Reasons for Tramadol/alcohol combination		
Euphoria	0(0.0%)	48(77.4%)
Pain Relief	0(0.0%)	4(6.5%)
Sex	0(0.0%)	36(58.1%)
Other drugs taken with alcohol		
Diclofenac	0(0.0%)	6(7.3%)
Felvin	0(0.0%)	8(9.8%)
MDMA	0(0.0%)	28(34.1%)
None		40(48.8%)
Reasons for other drugs		
Ecstasy	0(0.0%)	38(46.3%)
Sex	0(0.0%)	34(41.5%)
Pain Relief	0(0.0%)	10(12.2%)

Renal function parameters of test subjects were compared with control. There was a significant decrease in serum bicarbonate levels in test subjects (16.19±0.53), who had taken Tramadol and Alcohol combination within one week of sample collection in comparison with control (22.60±0.68; p=0.000). Serum urea was significantly higher in test subjects (38.03±2.08; p=0.000) than control (28.73±1.03). There was no significant difference in serum Potassium, Sodium, Chloride and Creatinine levels when compared with the control (p>0.05) (Table 2).

**Table 2:** Mean renal function parameters based on study participants groups *: Values are significantly different from control at P< 0.05

	Control	Test	t-test	p-value
Potassium (mmol/l)	3.71±0.04	3.62±0.05	1.553	0.123
Sodium (mmol/l)	135.90±0.48	131.45±0.48	0.658	0.512
Chloride (mmol/l)	94.83±1.54	92.19±1.61	1.182	0.240
Bicarbonate (mmol/l)	22.60±0.68	16.19±0.53	7.497	0.000*
Creatinine (umol/l)	62.27±1.75	69.06±2.97	-1.955	0.053
Urea (mg/dl)	28.73±1.03	38.03±2.08	-3.961	0.000*

**Figure 1:**
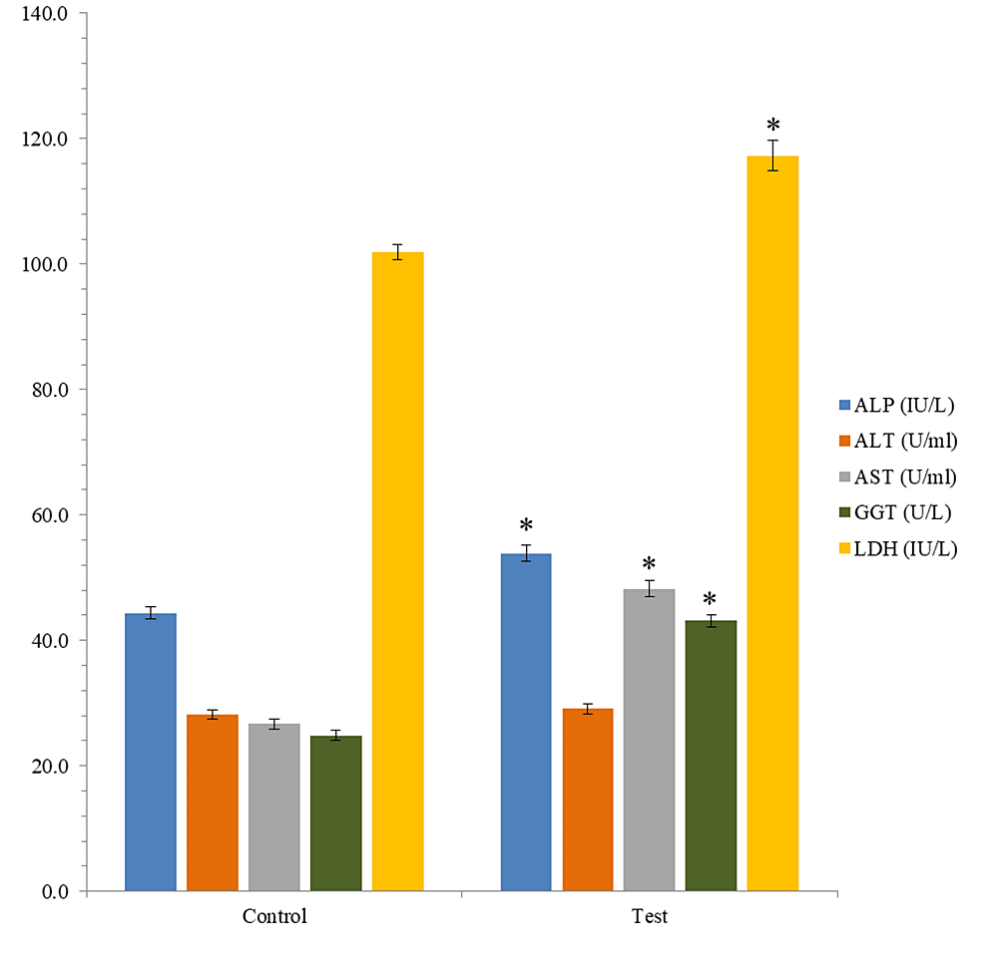
Liver enzymes in test subjects consuming Tramadol and Alcohol combination and non-alcohol and drug users (Control). *: Values are significantly different from control at P< 0.05. ALP: Alkaline phosphatase, ALT: Alanine Aminotransferase, AST: Aspartate Aminotransferase, GGT: Gamma glutamyl transferase, LDH: lactate dehydrogenase.

Alkaline phosphatase (ALP) was significantly raised in Test subjects (53.87±1.29) in comparison with the control (44.33±0.97; p=0.000), while there was no significant increase in alanine aminotransferase (ALT) in the test subjects (29.13±0.80) when compared with the control (28.17±0.76; p=0.39). Significantly higher levels of aspartate aminotransferase (AST) were observed for Test subjects (48.26±1.34) than in control subjects (26.70±0.80; p=0.000). GGT and lactate dehydrogenase (LDH) were significantly higher in Test subjects consuming Tramadol and Alcohol combination (43.13±1.02 and 117.29±2.45 respectively) than in control subjects (24.87±0.82; p=0.000 and 101.93±1.25; p=0.000, respectively) (Figure 1).

**Figure 2:**
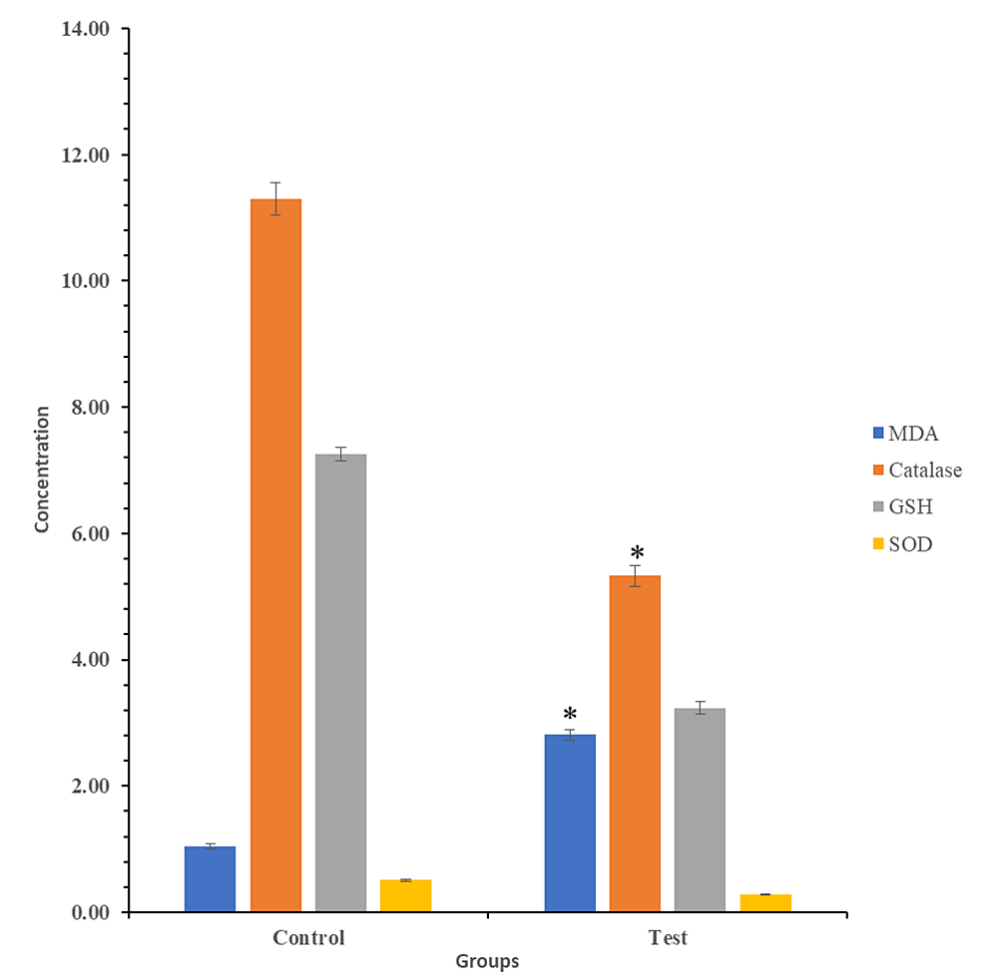
Oxidative stress markers of Test subjects consuming Tramadol and Alcohol combination and non-alcohol and drug users (Control). *: Values are significantly different from control at P< 0.05. MDA: malondialdehyde, GSH: reduced glutathione, SOD: superoxide dismutase

Malondialdehyde (MDA) which is produced by the peroxidation of membrane polyunsaturated fatty acids and an important marker of oxidative stress, was significantly increased in Test subjects consuming Tramadol and Alcohol combination (2.81±0.08) in comparison with the control non-drug alcohol-drug users (1.04±0.04; p=0.000). A significant decrease in serum antioxidants level was observed in test subjects. Serum catalase, glutathione and superoxide dismutase were significantly reduced in test subjects consuming Tramadol and Alcohol combined (5.33±0.16, 3.23±0.10 and 0.29±0.01 respectively), comparison with higher serum levels of catalase, glutathione and superoxide dismutase seen in control subjects (11.29±0.25, 7.26±0.10, and 0.51±0.01 respectively; p<0.05) (Figure 2).

**Figure 3:**
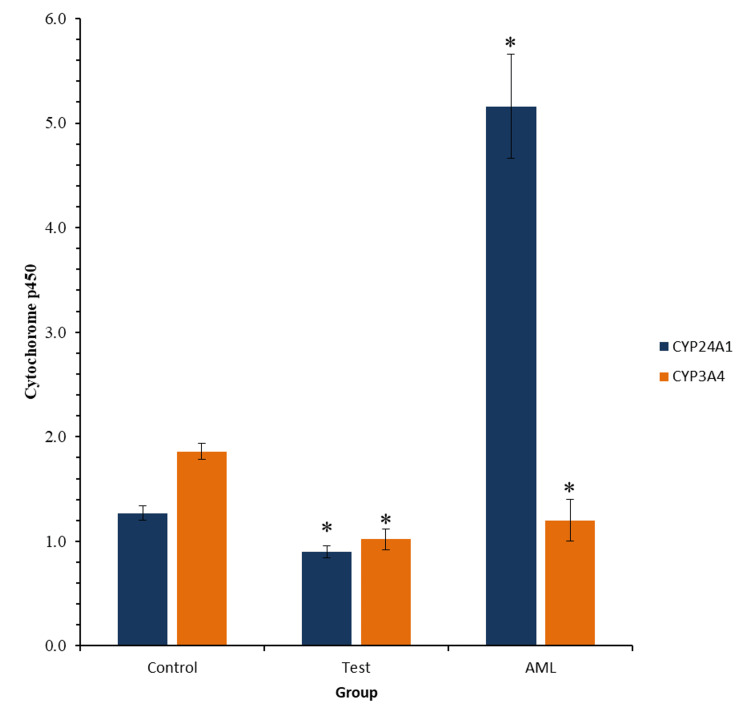
Serum CytochromeP450 24A1 (CYP24A1) and CytochromeP450 3A4 (CYP3A4) of subjects consuming Tramadol and Alcohol combination (Test) compared with non-Alcohol and Tramadol users as normal control and subjects with acute myeloid leukemia (AML) as abnormal or positive control. *: Values are significantly different from control at P< 0.05.

The Cytochrome P450 enzymes are a family of enzymes known to be involved in the metabolism of carcinogens and drugs, and other toxicants. Serum level of Cytochrome P450 24A1, which is a subfamily of Cytochrome P450 enzymes, was significantly lower in Test subjects (subjects consuming Tramadol and Alcohol combination) (0.90±0.06; p=0.01), and significantly threefold higher in subjects with AML (5.16±0.5; p=0.000), when compared with values of non-drug users that served as normal control (1.27±0.07). Subjects with AML also had significantly higher Cytochrome P450 24A1 values than Test subjects (p=0.000). Serum level of Cytochrome P450 3A4 was significantly lower in Test subjects (subjects consuming Tramadol and Alcohol combination) (1.02±0.10; p=0.000), and also significantly lower in subjects with AML (1.20±0.2, p=0.04). Although Cytochrome P450 3A4 level in AML was higher than in Test subjects consuming Tramadol and Alcohol combination, the difference was not statistically significant (p=0.76) (Figure 3).

**Figure 4:**
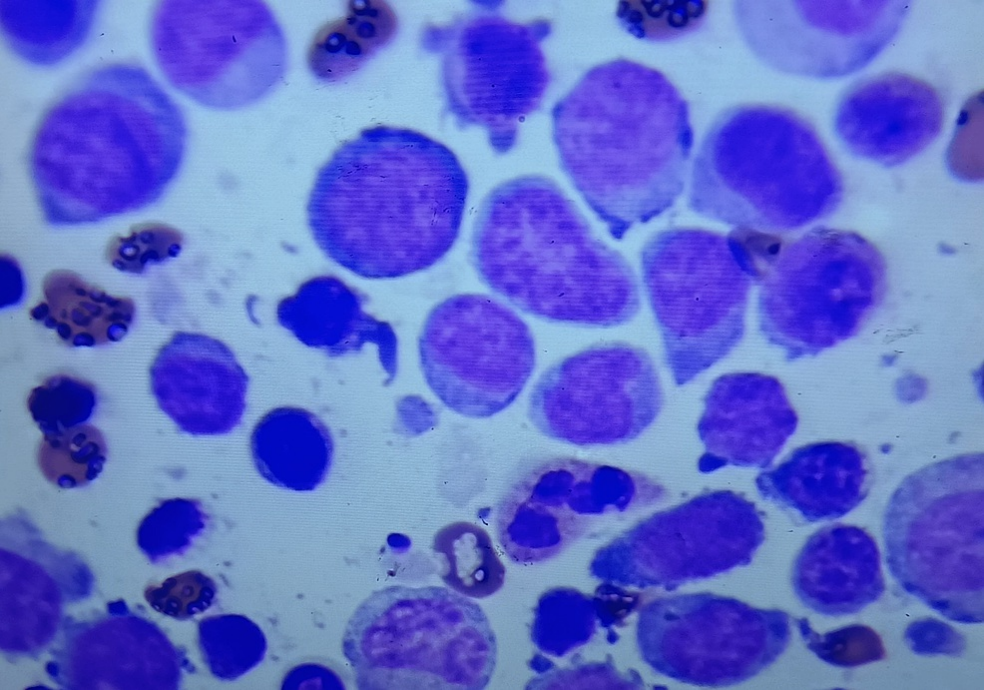
Bone marrow aspirate from positive control subjects with acute myeloid leukemia (x100 objective).

Photomicrograph of Romanowsky stained bone marrow aspirates from a positive control subject showing AML with blast cells (Figure 4).

## Discussion

Analgesics misuse, especially Tramadol abuse is evolving into a health crisis. The recent menace of trafficking of pharmaceutical opioids especially Tramadol into Nigeria usually for non-medical purposes has strengthened concerns about its abuse [4,9]. A recent study identified Tramadol as the most misused drug after cannabis in Nigeria. As of 2017, the rate of Tramadol abuse in Nigeria was put at 4.6 million people [10].

Our study showed that drug users that combine Tramadol with alcohol in the study population were largely university undergraduates (75.6% of participants) while the other 12.2% of subjects were artisans. 77.4% of study participants reported euphoria as their reason for combining Alcohol and Tramadol, and 6.5% claimed it was for faster pain relief and enhanced sexual performance, or prolong penile erection was the response of 58.1% of the test subjects. This agrees with a previous study [5] in which young adults the majority of whom were University undergraduates used a cocktail of Tramadol, Cannabis, and Vodka, a cocktail that was referred to as “Gutter water.” Co-abuse of Tramadol and Alcohol among secondary (high) school students in urban parts of Nigeria such as Oyo state and Enugu [4,9], and other parts of West Africa such as Cameroon [11].

As observed in this study, reasons given by respondents for consumption of Tramadol and Alcohol combinations were; to have the feeling of ecstasy, euphoria, and prolonged penile erection (aphrodisiac effect), which aligns with a previous study by Dumbili et al., which reported that the major motivation for Tramadol-Alcohol combination was for heightening and prolonging intoxication and sense of pleasure (euphoria) [5]. A study by the United Nations Office on Drugs and Crime, also reported that euphoria/ecstasy and an increase in sexual performance were major motives for Tramadol abuse [10]. All respondents in our study reported anxiety relief and good mood with high doses of Tramadol.

Findings from our study showed the risk of renal impairment, as our findings showed a decrease in blood electrolytes, a significant reduction in bicarbonate, and a significant increase in blood urea among the test group using Tramadol and Alcohol concomitantly. Several mechanisms that have been suggested for Nephrotoxicity accompanying Tramadol abuse in humans include rhabdomyolysis, decreased renal perfusion, secondary amyloidosis [12], and interstitial inflammation [13].

The liver is the primary site for the metabolism of drugs and alcohol [14-16]; hence, it is the organ most affected by their toxicity. Liver enzymes which include aspartate amino transferase, alanine amino transferase, gamma-glutamyl transferase, alkaline phosphatase, and lactate dehydrogenase were elevated in the test group using Alcohol and Tramadol concomitantly. Similar studies by Owoade et al. and Elkhateeb et al. have reported the hepatotoxic effect of Tramadol and Alcohol combined in experimental animals [3,17]. Studies by Elmanama et al. and Youssef et al. have also reported the Hepatotoxic effect of Tramadol and Alcohol concomitant use in humans [18,19].

Elevated malondialdehyde activity, decreased catalase, superoxide dismutase, and reduced glutathione in test participants misusing Tramadol and Alcohol concomitantly, was an indication of lipid peroxidation and oxidative stress. This reveals that Alcohol and Tramadol combination induces hepatic damage through oxidative stress and the generation of free radicals. Studies have revealed that oxidative stress causes hepatic damage by triggering irreversible alteration of lipids, proteins, and DNA contents, and modulating pathways that control normal biological functions [20,21].

It has been postulated that drugs that induce CYP3A4 protein activity can increase the metabolism of Tramadol to inactive metabolites, hence reducing its analgesic effect [22]; from this current study, chronic concomitant administration of alcohol along with Tramadol reduced CYP3A4 activity, also AML reduced CYP3A4. Reports have shown that CYP3A4 is involved in the metabolism of approximately 50% of commercially available drugs [23], and this broad range of metabolism may be the reason for CYP3A4 generating significant reactive oxygen species (ROS) during drug metabolism, due to its flexible active site which may give space for high rates of reaction uncoupling [24,25].

Cytochrome P450 24A1 (CYP24A1) is a mitochondrial protein that has critically been reported to be responsible for the deactivation of the biologically active form of vitamin D, 1, 25 (OH)2 D3 [26,27]. Concomitant use of Alcohol and Tramadol resulted in a significant decrease in CPY24A1 protein levels. In the AML patients used as a positive control for the human study, a three- to fivefold increase in CPY24A1 protein level was recorded. In a recent study, increased expression of the CYP24A1 gene and serum levels of the CYP24A1 protein was significantly elevated in AML subjects [28]. Emerging evidence from studies is linking some cancers with decreased serum vitamin D levels because, in addition to the maintenance of skeletal functions and mineral homeostasis, the active vitamin D metabolite Calcitriols (1, 25-Dihydroxyvitamin D(3), 1,25 (OH)(2)D(3)) also possess anti-Proliferative, apoptotic, differentiation-inducing, and Immunomodulatory effects on cancer cells [29]. There is an increasing incidence of cancer among young people in Nigeria [30], and also high alcohol use among this age demography, hence the need for more extensive study on the impact of chronic alcohol use which can induce severe CYP24A1 protein expression, cancer induction and proliferation of cancer cells. The possible use of Calcitriol as a prophylactic and management strategy has to be further elucidated. CYP3A4 and CYP24A1 protein expression was not determined for study participants that admitted to concomitant use of alcohol and drugs such as MDMA, Felvin, and diclofenac, as multiple drug use (Tramadol and any of the abused drugs) concomitantly with alcohol could have affected our study outcome. This is one limitation of our study. The role of genes that have an impact on the risk for alcoholism or related traits may affect CYP24A1 expression. This was not considered in our study and amounts to one of our study limitations.

## Conclusions

From our study, it has been substantiated that the rate of concomitant use of Tramadol and Alcohol among young people in Nigeria is high, and our findings of hepatotoxicity posed by the continuous use of this cocktail are sufficient evidence to disabuse the minds of the public on this unhealthy practice. The involvement of Cytochrome p450 24A1 and CytochromeP450 3A4 in the metabolism of Tramadol and Alcohol combination has been revealed in our study.
